# Supplementary material for: Unifying host-associated diversification processes using butterfly–plant networks
Source: Nat Commun. 2018 Dec 4;9:5155. doi: 10.1038/s41467-018-07677-x (PMC6279759; doi:10.1038/s41467-018-07677-x)
Supplement: Supplementary file 1 — Description of Additional Supplementary Files [file 41467_2018_7677_MOESM1_ESM.pdf]

Description of Additional Supplementary Files

File Name: Supplementary Software 1
Description: R script to simulate theoretical diversification models and generate networks.
