# Supplementary material for: Unifying host-associated diversification processes using butterfly–plant networks
Source: Nat Commun. 2018 Dec 4;9:5155. doi: 10.1038/s41467-018-07677-x (PMC6279759; doi:10.1038/s41467-018-07677-x)
Supplement: Supplementary file 4 — Supplementary Information [file 41467_2018_7677_MOESM4_ESM.pdf]

Supplementary Information

Unifying host-associated diversification processes using butterfly-plant networks

Mariana P Braga, Paulo R Guimarães Jr, Christopher W Wheat, Sören Nylin and Niklas Janz

1 Network of interactions between Nymphalidae genera and plant families

Supplementary Methods – To build the Nymphalidae genera-plant families network we used the host use dataset sampled by Hamm and Fordyce (2015), which includes 982 interactions between 380 butterfly genera and 136 plant families with know phylogenetic position (Supplementary Figs. 7-8). This network is both significantly nested and modular (Supplementary Table 1). Butterflies and plants were grouped in 8 modules by the optimisation algorithm (Supplementary Fig. 7).

Hamm, C. A. & Fordyce, J. A. Patterns of host plant utilization and diversification in the brush-footed butterflies. *Evolution* **69,** 589–601 (2015).

2 Complete summary of the analysis of all simulated and empirical networks in the study

Supplementary Table 1 – Nestedness, modularity and number of modules for every network in the study. P-values and Z-scores are relative to networks generated by the CE null model, and therefore can be used to compare the degree of nestedness and modularity between networks. Values higher than expected by null models (alpha = 0.05) are in bold.

	Nestedness			*Modularity*			
	NODF	*p*	*z*-score	M	# modules	*p*	*z*-score
R5V0	0	1	-9.20	**0.77**	**6**	**< 0.01**	**8.21**
R4V1	1.74	1	-7.60	**0.76**	**6**	**< 0.01**	**6.83**
R3V2	4.82	1	-7.12	**0.64**	**7**	**< 0.01**	**4.62**
R2V3	9.48	1	-4.72	**0.52**	**7**	**< 0.01**	**3.77**
R1V4	13.72	0.78	-0.77	**0.42**	**7**	**0.01**	**2.83**
R0V5	**18.8**	**< 0.001**	**3.78**	0.35	7	0.12	1.09
Random	22.62	0.77	-0.71	0.23	7	0.81	-0.79
Nymphalidae - plant orders	**13.09**	**< 0.001**	**8.27**	**0.58**	**10**	**< 0.01**	**3.96**
Nymphalidae - plant families	**11.79**	**< 0.001**	**16.58**	**0.58**	**8**	**< 0.01**	**5.55**
Pieridae - plant families	**14.23**	**< 0.001**	**3.9**	**0.66**	**10**	**0.03**	**1.96**
Uniform evolution	10.55	0.26	0.59	**0.54**	**6**	**0.02**	**1.96**

***3** Supplementary figures*

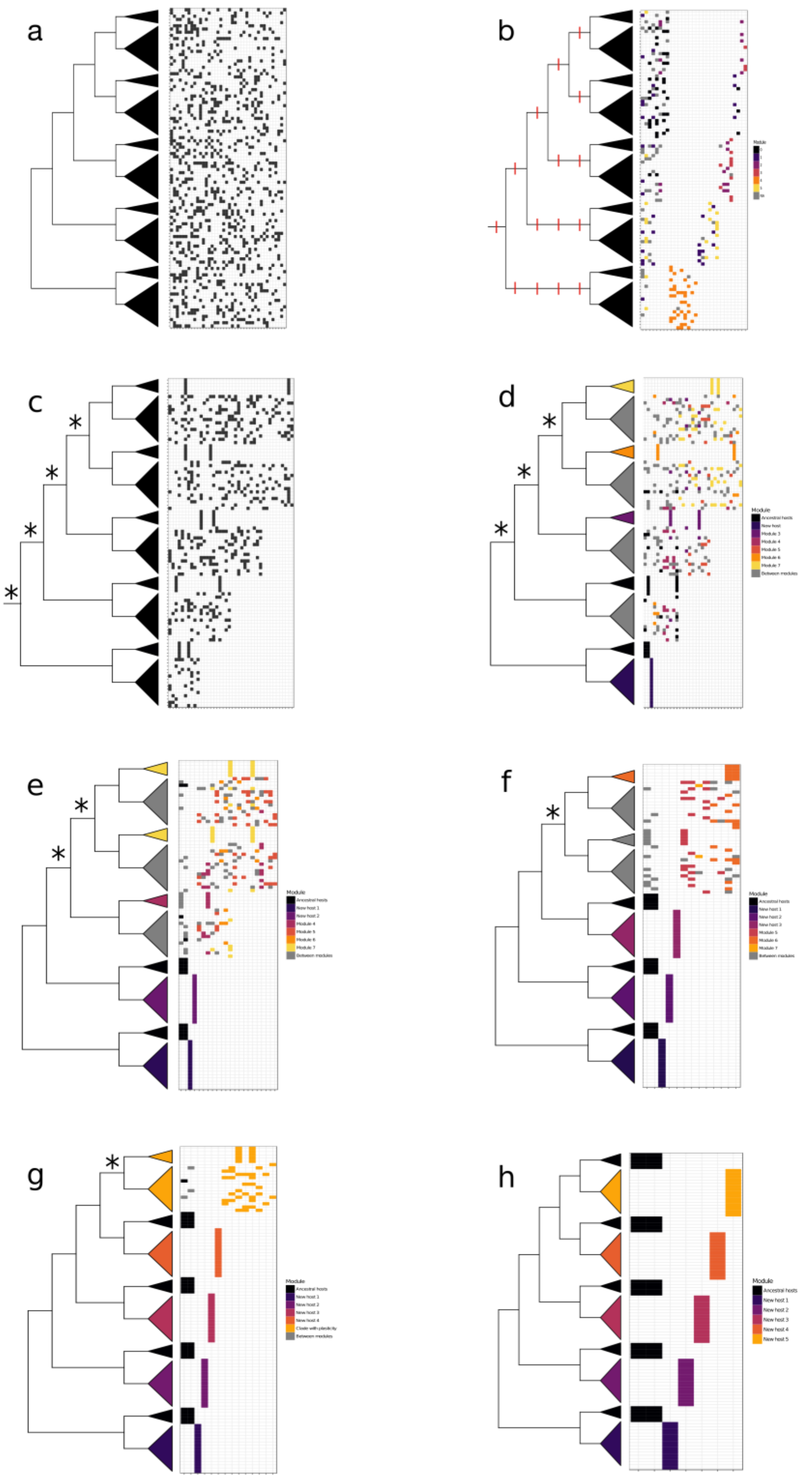

Supplementary Figure 1 – Simulated insect-plant networks. In each panel, insect phylogeny on the left and network on the right, showing realised interactions between insects (rows) and host plants (columns). Interactions and clades are coloured by modules. Red lines in panel b indicate addition of two hosts to the fundamental repertoire. Asterisks indicate addition of 10 hosts to the fundamental host repertoire (start of the variability scenario). Networks simulated following (a) random interactions, (b) uniform evolution scenario, (c) the variability scenario, (h) the radiation scenario, and (d-g) mixtures of the radiation and the variability scenarios.

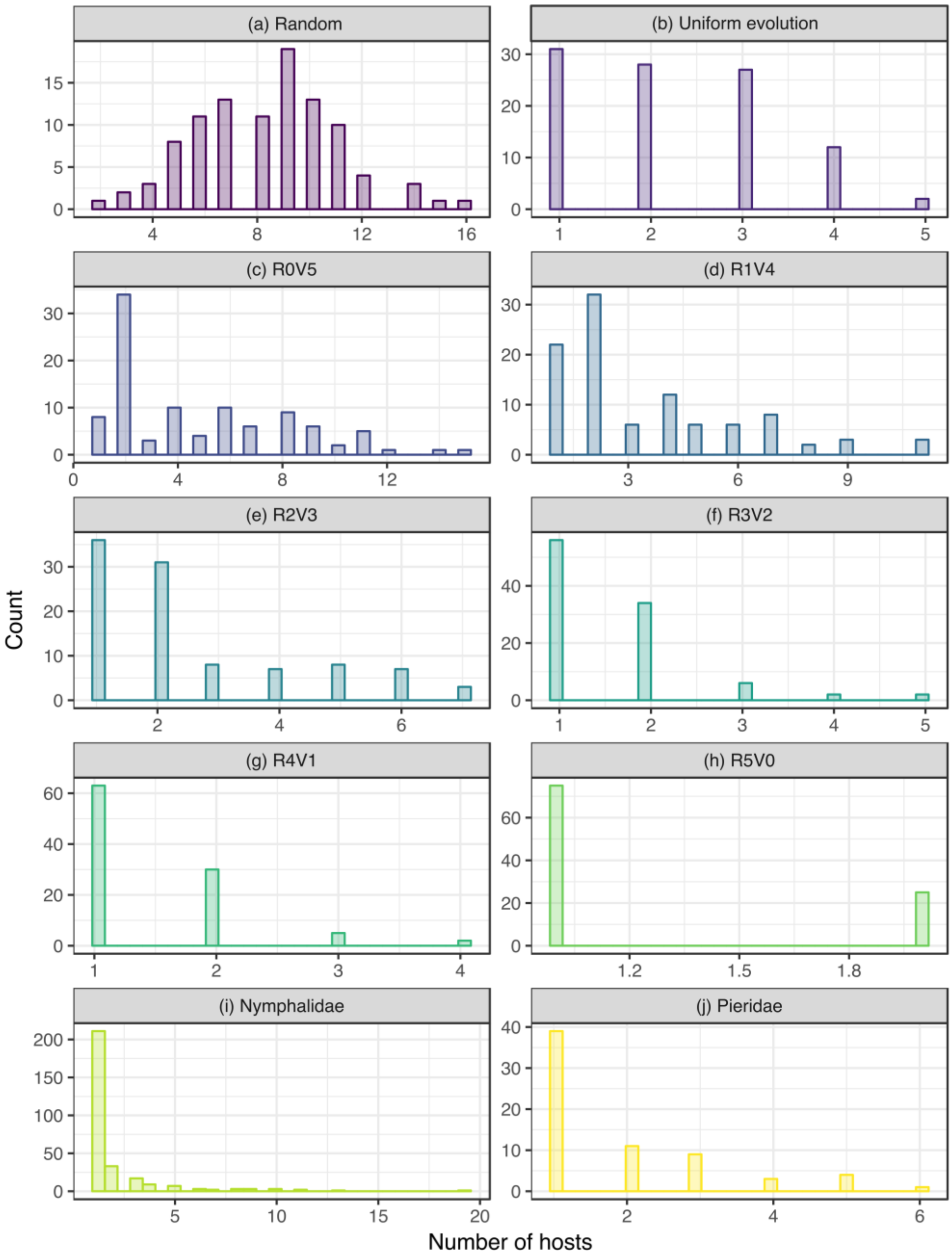

Supplementary Figure 2 – Host range distribution of simulated and empirical networks. Each panel shows the distribution for (a) random network, (b) uniform evolution scenario, (c-h) variability-radiation gradient (letters are consistent with Supplementary Figure 1 and codes are consistent with Figure 1), (i) Nymphalidae network, and (j) Pieridae network. Please note that axes are not scaled across panels.

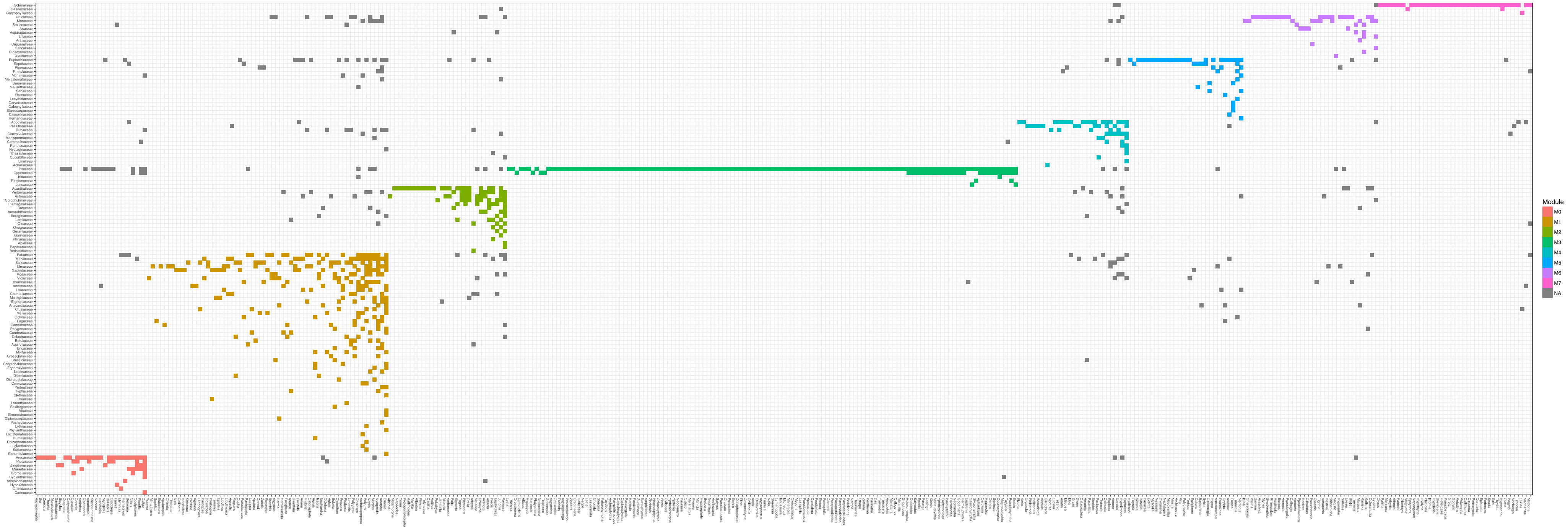

Supplementary Figure 3 - Nymphalidae-plant family network. Nymphalidae genera in columns and host plant families in rows. Rows and columns sorted to emphasise modular affinity (order of modules is arbitrary). Each colour shows interactions within a module, and grey cells are interactions between modules.

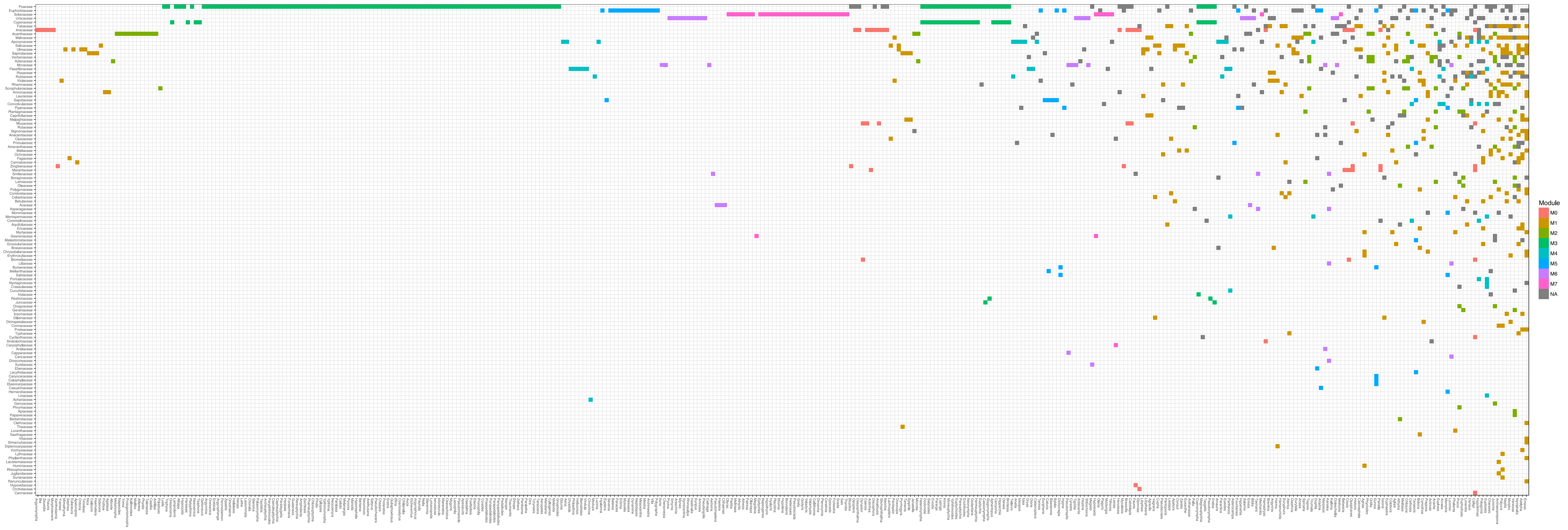

Supplementary Figure 4 - Nymphalidae-plant family network. Nymphalidae genera in columns and host plant families in rows. Rows and columns sorted to emphasise nestedness, ordered from the upper right corner according to descending number of interactions. Colours also represent modules and are consistent with Supplementary Figure 3.

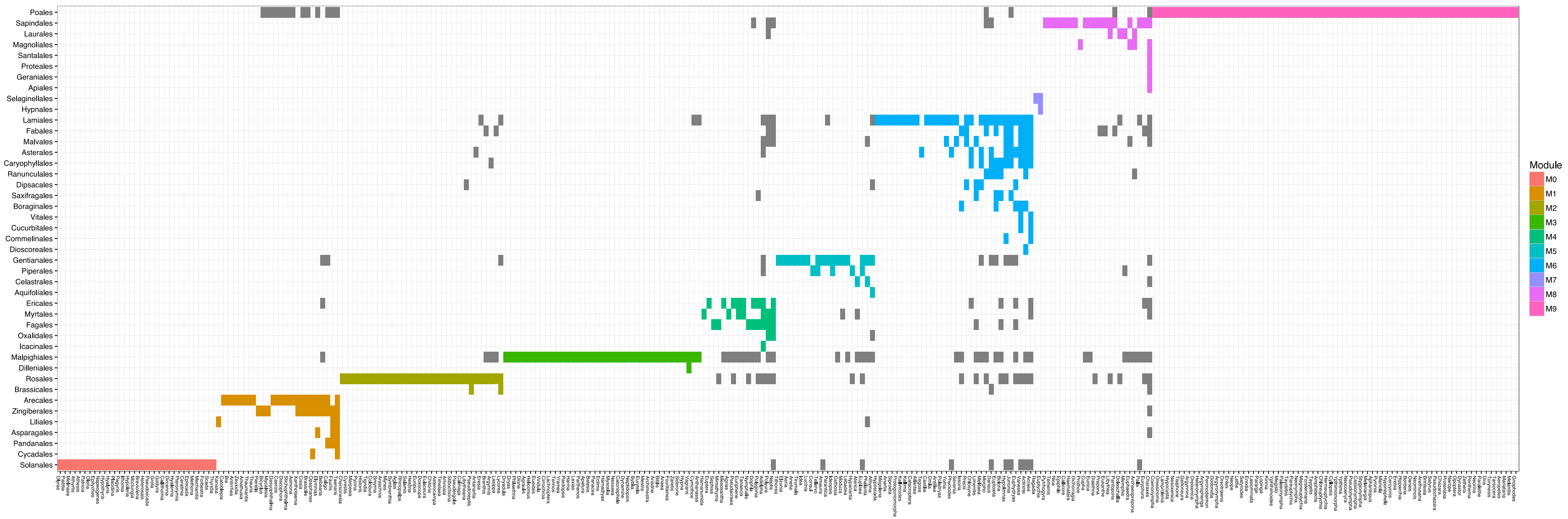

Supplementary Figure 5 - Nymphalidae-plant order network. Nymphalidae genera in columns and host plant orders in rows. Rows and columns sorted to emphasise modular affinity (order of modules is arbitrary). Each colour shows interactions within a module, and grey cells are interactions between modules.

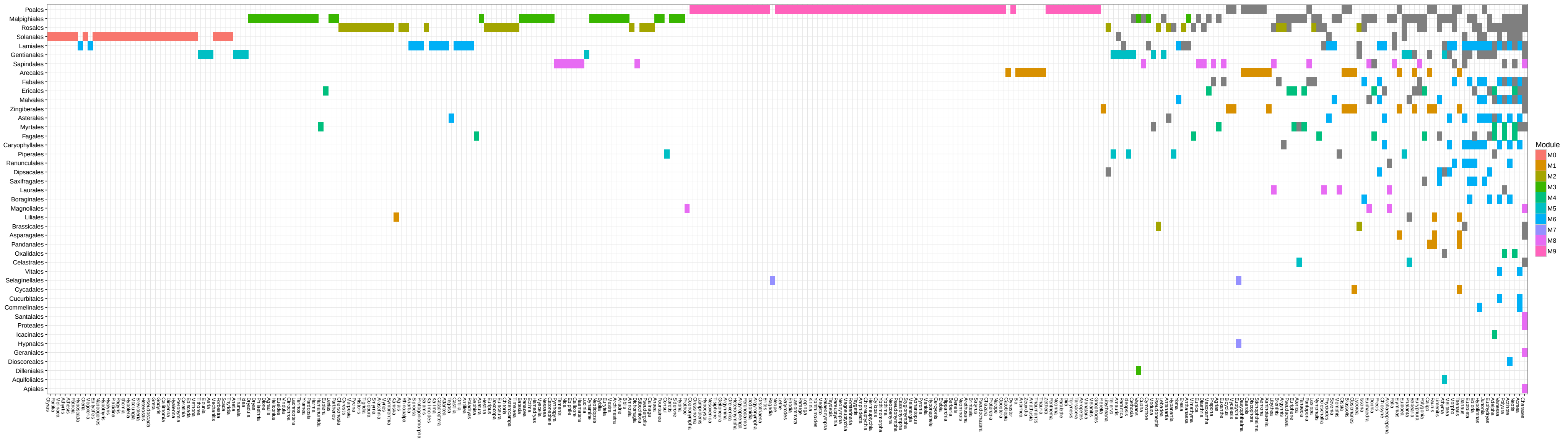

Supplementary Figure 6 - Nymphalidae-plant order network. Nymphalidae genera in columns and host plant orders in rows. Rows and columns sorted to emphasise nestedness, ordered from the upper right corner according to descending number of interactions. Colours also represent modules and are consistent with Supplementary Figure 5.

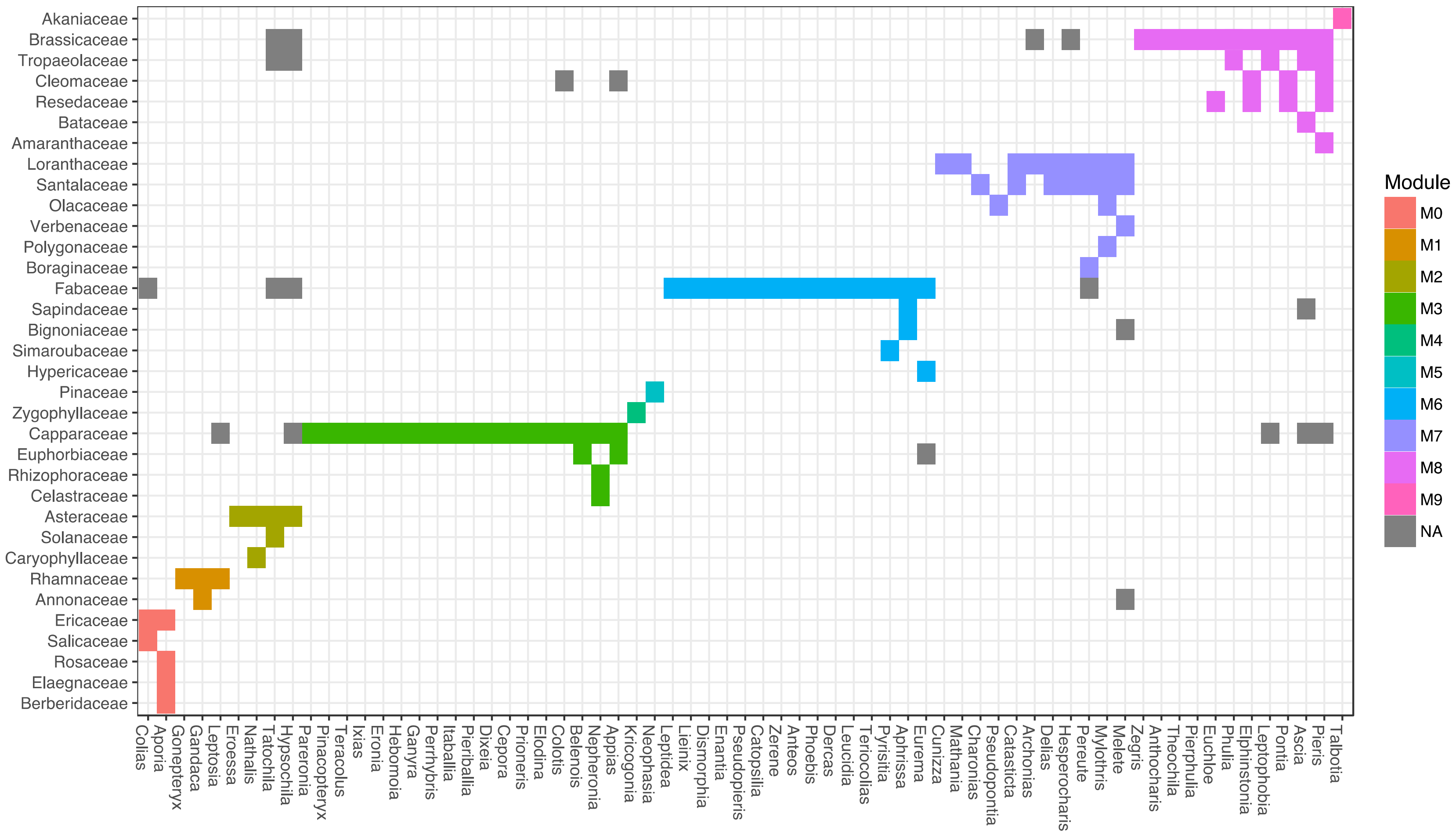

Supplementary Figure 7 - Pieridae-plant network. Pieridae genera in columns and host plant families in rows. Rows and columns sorted to emphasise modular affinity (order of modules is arbitrary). Each colour shows interactions within a module, and grey cells are interactions between modules.

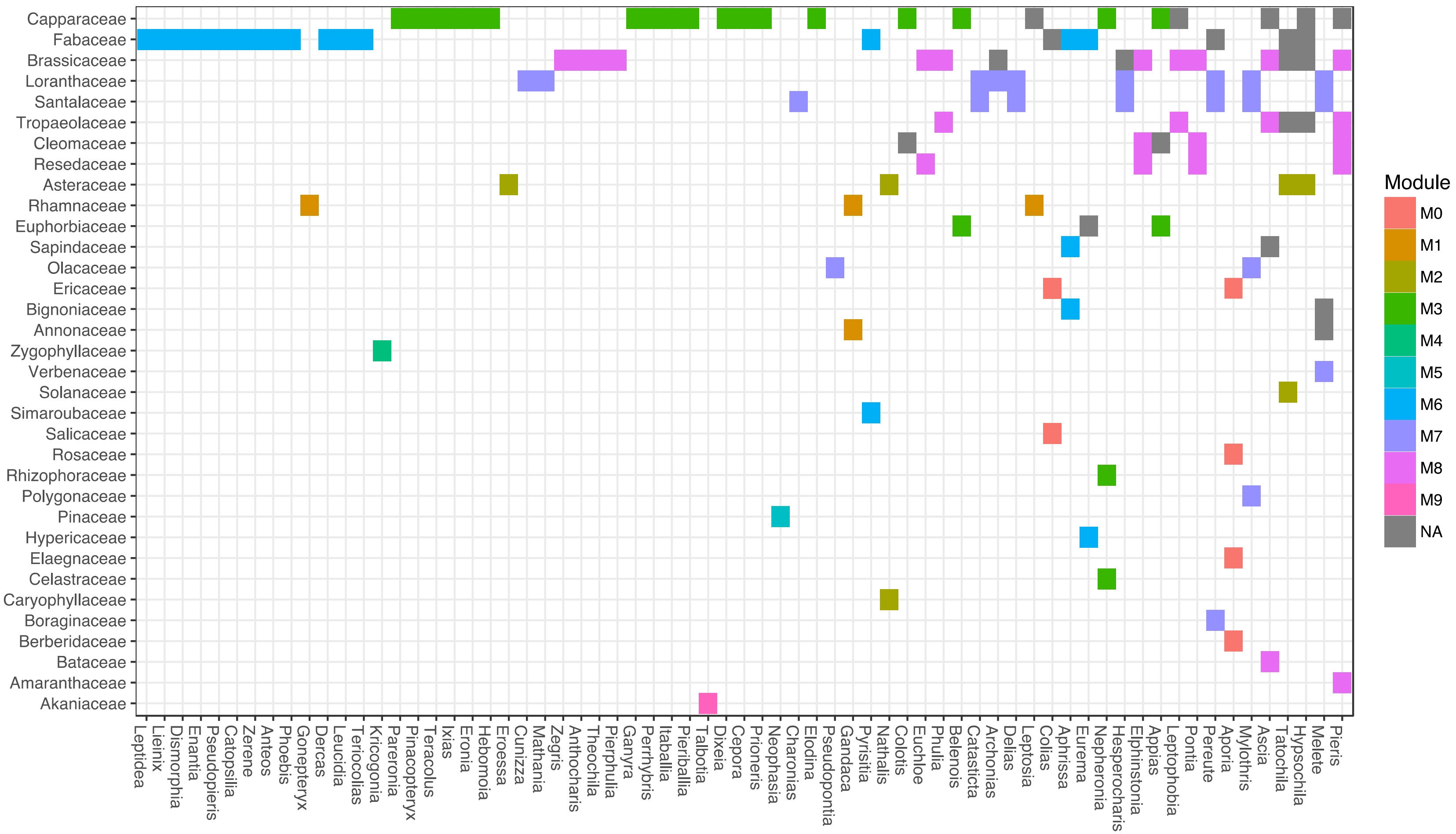

Supplementary Figure 8 - Pieridae-plant network. Pieridae genera in columns and host plant families in rows. Rows and columns sorted to emphasise nestedness, ordered from the upper right corner according to descending number of interactions. Colours represent modules and are consistent with Supplementary Figure 7.
